# Potential of marine dinoflagellates *Amphidinium carterae* and *Prorocentrum minimum* as fatty acids producers: A comparative transcriptome and fatty acid profiling study

**DOI:** 10.71150/jm.2604005

**Published:** 2026-06-30

**Authors:** Han-Sol Kim, Su-Hwa Lee, Zhun Li, Hah Young Yoo, Jang-Seu Ki

**Affiliations:** 1 Department of Life Science, Sangmyung University, Seoul 03016, Republic of Korea; 2 Institute of Natural Science, Sangmyung University, Seoul 03016, Republic of Korea; 3 Biological Resource Center/Korean Collection for Type Cultures (KCTC), Korea Research Institute of Bioscience and Biotechnology, Jeongeup 56212, Republic of Korea

**Keywords:** dinoflagellate, *Amphidinium*, *Prorocentrum*, fatty acid (FA), omega-3, lipid droplet-associated hydrolase (LDAH)

## Abstract

Marine dinoflagellates are gaining attention as sustainable bioresource for polyunsaturated fatty acids (PUFAs), particularly omega-3 such as eicosapentaenoic acid (EPA) and docosahexaenoic acid (DHA). In the present study, we analyzed the FAs and transcriptomic profiles of marine dinoflagellates *Amphidinium carterae* (D-044) and *Prorocentrum minimum* (D-127) to evaluate their potential as FAs producers. Gas chromatography-FA methyl ester (GC-FAME) analysis showed that *A. carterae* is a superior omega-3 producer, yielding a total FA content of 67.6 mg/g DW. DHA accounted for 26.7% of the total FAME profile, which is significantly higher than that of *P. minimum* (18.1 mg/g DW; DHA 13.1%). Gene Ontology (GO) annotation revealed genes related to FAs and lipid metabolism in *A. carterae* (1,217 genes) and in *P. minimum* (2,317 genes), which provide a molecular basis for dinoflagellates with high lipid productivity. Notably, three lipid droplet-associated hydrolase (*LDAH*) genes with diverse evolutionary origins were identified from *A. carterae*. These findings suggest a potential expansion of the genetic repertoire related to lipid storage and metabolism, highlighting *A. carterae* and *LDAH* as candidates for future biotechnological applications and microalgal metabolic engineering.

## Introduction

Microalgae are a group of single-cell photosynthetic organisms that occur in diverse aquatic systems from freshwater to the ocean (Singh and Saxena, 2015). Among those, dinoflagellates are one of the largest groups of marine microalgae in terms of species number (Guiry and Guiry, 2026; Taylor et al., 2008). They play a crucial role in primary production and coral reef formation in marine environments (Sampayo et al., 2007). Interestingly, dinoflagellates are known to have complex biosynthetic pathways that yield distinctive metabolites, such as macrolides. For example, several species of *Amphidinium* and *Prorocentrum* can synthesize amphidinolides (Kobayashi et al., 2003) and prorocentrolides (Amar et al., 2018). Moreover, some dinoflagellates can produce high levels of fatty acids (FAs), raising commercial interest as aquafeeds and alternatives to fish oil for nutraceuticals (Leblond and Chapman, 2000; Remize et al., 2020).

Fatty acids (FAs) are fundamental components of cellular membranes and storage lipids and serve as precursors to a wide range of metabolites (Ali and Szabó, 2023; De Carvalho and Caramujo, 2018). Polyunsaturated FAs (PUFAs), notably omega-3 and omega-6, play important roles in controlling membrane properties, cell signaling, and inflammation (Calder, 2001). In particular, eicosapentaenoic acid (EPA; C20:5n-3) and docosahexaenoic acid (DHA; C22:6n-3) are representative omega-3, functioning as key regulators of membrane fluidity and cellular defense against stresses (Gorjão et al., 2009; Hu et al., 2024). Also, EPA and DHA are associated with benefits in aging, neurodevelopment, and inflammatory disorder improvements (Hu et al., 2024; Uauy and Dangou, 2006). In this regard, the demand for omega-3 continues to increase, and the global omega-3 market was valued at $2.1–22.8 billion in 2024 (FAO, 2020; Research and Markets, 2021). Their production heavily depends on marine fisheries (Glencross et al., 2025), however, fish oil supply is limited by overfishing and sustainability issues (Byreddy et al., 2019; Tocher et al., 2019). Consequently, microalgae have emerged as sustainable and alternative producers of omega-3 production (Russo et al., 2021).

The dinoflagellates *Amphidinium* and *Prorocentrum* are pivotal microalgae in marine ecosystems and are also highly valued as emerging resources for omega-3. Their exceptional capacity to synthesize and store PUFAs positions them as promising and sustainable sources for nutraceutical and pharmaceutical applications (Orefice et al., 2023). However, studies on the omega-3 production potential of dinoflagellates remain fragmented compared to other industrial strains *Schizochytrium* sp. and *Crypthecodinium cohnii*. For example, previous studies were often limited to FAs profiling under diverse culture conditions such as temperature (La Motta et al., 2024), light intensity (Molina-Miras et al., 2022; Yin et al., 2023), nutrients (Jiang et al., 2014; Weng et al., 2014), and diverse stresses (Kusumaningtyas et al., 2017; Schmitz et al., 2025). Moreover, the intrinsic omega-3 metabolic flux and storage at the molecular level are poorly understood.

Recent advances in high-throughput sequencing have revealed that the mechanisms involved in FAs biosynthesis in microalgae are complex, with numerous isoforms and unique metabolic flux (Schmitz et al., 2025). Nevertheless, research on dinoflagellates has been limited to transcriptional changes in response to culture conditions and toxins biosynthesis (Bui et al., 2021; Kim et al., 2021, 2023; Muhammad et al., 2026; Wang et al., 2021a). Accordingly, functional genes participating in lipid synthesis in dinoflagellates remain poorly characterized, which is a challenge to solve for future efficient metabolic engineering and commercial process optimization. Therefore, in the present study, we investigated marine dinoflagellates *Amphidinium carterae* and *Prorocentrum minimum*, focusing on FAs profiles and transcriptome analysis. Firstly, we observed the lipid droplet formation and quantified FAs using gas chromatography-FA methyl ester (GC-FAME) analysis to compare the profiles. Then, we analyzed transcriptome to identify genes related to FAs metabolisms and lipid droplet formation. The tested species are harmful algal bloom causative dinoflagellates that have been studied for large-scale cultivation and natural compounds production.

## Materials and Methods

### Cell isolation and strain culture

The strains of *A. carterae* (D-044) and *P. minimum* (D-127) used in this study were obtained from the Korea Marine Microalgae Culture Center (Pukyong National University, Korea). Each strain was originally isolated from a single vegetative cell collected from the coastal waters of South Korea, and cultured in f/2 medium without silicate (Ryther and Guillar, 1962). The strains were cultured at 20°C and 65 μmol photons/m^2^/s of photon flux density under a 12:12 h light–dark cycle. The cells were observed using both light and epifluorescence microscopy (Carl Zeiss Axioskop, Germany) equipped with a ProgRes^®^ CF scan CCD camera (Jenoptik, Germany).

### Cell density and size measurements

Changes in cell density was evaluated using a plankton-counting chamber (Matsunami Glass, Japan) under a light microscope (Carl Zeiss Axioskop) to examine the growth patterns of *A. carterae* and *P. minimum*. Each sample was fixed by adding Lugol’s solution (final concentration of 1%) and all counts were performed independently in triplicate. Additionally, the average cell size was analyzed using an Auto T4 Cellometer^TM^ (Nexcelom Biosciences, USA).

### Nile red staining

Nile Red fluorescent dye (CAS No. 7385-67-3; Sigma-Aldrich) was used to stain and observe the neutral lipids in the test species. The stock solution was dissolved as 0.1 mg/ml in acetone (320110; Sigma-Aldrich). Fresh cells (1 × 10^6^ cells/ml) from the exponential, stationary, and decline phases were stained with a final concentration of 1.0 μg/ml. Samples were incubated in darkness for 7 min, and washed three times with fresh f/2 medium. The orange fluorescent was observed using a fluorescence microscope (Carl Zeiss Axioskop) to determine the lipid droplet production.

### Fatty acid methyl ester (FAME) analysis

For FA analysis, 5–10 L of the cultures in the exponential phase were harvested by centrifugation at 3,000 × g for 10 min. The supernatant was removed, and the cell pellets were collected and lyophilized through freeze-drying. The freeze-dried biomasses were then precisely weighed to determine the dry cell weight (DCW), which served as the starting materials of FA extraction and analysis. Lipid extractions and transesterification to FAME were performed using 5% acetyl chloride (CH_3_COCl) in methanol, following the method of Lepage and Roy (1986). FAME profiles of the samples were analyzed using a Shimadzu GC 2010 Plus gas chromatograph (Shimadzu, Japan) with a DB-23 capillary column (Agilent J&W) and a flame ionization detector (FID). Methyl heptadecanoate and a Supelco 37 component FAME Mix (CRM47885, Merck, Germany) were used as the internal standard (IS) and FAME standards, respectively. Detailed analytical conditions were followed previously published methods (Kang et al., 2025). The FAs profile was categorized into saturated FAs (SFAs), monounsaturated FAs (MUFAs), polyunsaturated FAs (PUFAs), and unidentified FAs (UnFAs). The total FAs contents were calculated as mg/g of initial freeze-dried DCW. The FAME content was calculated using the following equation.


$$\;FAME\;content\left(\%\right)=\frac{\left(\frac{FAME\;area-IS\;area}{IS\;area}\right)\left(IS\;concentration\right)\left(IS\;dilution\;factor\right)}{DCW}\times 100$$

### RNA extraction and cDNA synthesis

To gather as much gene information as possible, *A. carterae* and *P. minimum* cells under diverse conditions were harvested; thermal shock (30°C for 12 and 24 h), exposure to UV irradiation (20 min) and toxic contaminants (0.5 mg/L CuSO_4_, 0.5 mg/L CuCl_2_, 0.5 mg/L NaOCl, and 0.5 mg/L NiSO_4_ for 6 and 24 h), and different growth stages (lag, exponential, and declining phases). About 200 ml of each culture were centrifuged at 3,000 × g for 10 min, and the pellets were mixed with 1 ml of RiboEx solution (Geneall Biotechnology Co. LTD., Korea) and stored at −80°C until the extraction. The total RNA was extracted using GeneAll^®^ Hybrid-R^TM^ miRNA Kit (Geneall) following to the manufacturer’s instructions. RNA quality and quantity were determined using an Agilent 2100 Bioanalyzer (Agilent, USA). The complementary DNA (cDNA) was synthesized using the Illumina^®^ TruSeq^TM^ RNA Sample Preparation Kit (RS122-2001, Illumina Inc., USA). Sequencing was performed using Illumina HiSeq 2500 (Illumina Inc.).

### Transcriptome assembly and functional annotation

The raw sequence data was deposited in the National Center for Biotechnology Information (NCBI) Sequence Read Archive (SRA) database (BioProject accession no. PRJNA14639770). The quality of the raw reads was checked with FastQC_v0.11.7 (http://www.bioinformatics.babraham.ac.uk/projects/fastqc/). The reads were cleaned and trimmed by removing adaptor and low-quality reads using Trimmomatic v.0.38. The reads were then assembled using Trinity software and the assemblies were assigned contig codes. Additionally, the CD-HIT program v.4.6 was used for sequence clustering with a default setting to reduce sequence redundancy. All contigs were annotated against public databases gene ontology (GO) with an E-value default cutoff of 1.0E-5. To minimize redundancy of GO annotation terms across the biological process (BP), molecular function (MF), and cellular component (CC) categories, gene assignments were prioritized in the order of BP, MF, and CC. Furthermore, sub-annotations were consolidated into the top categories related to the lipid and fatty acid metabolism.

### Identification of *LDAH* genes and phylogenetic analysis

Identification of lipid droplet-associated hydrolase (*LDAH*) gene candidates was selected through filtering the annotated transcript to isolate contigs containing conserved domain. The selected contigs were then subjected to a BlastX search to exclude sequences derived from potential contaminations (Bacteria and Fungi). Subsequently, the contigs showing the best hits (E-value < 1.0E-5) within the microalgae or dinoflagellates taxonomy group were retained and designated as the final list of *LDAH* for subsequent analysis. Amino acid (aa) sequences of LDAH identified from *A. carterae* and *P. minimum* were used for the phylogeny analysis. The sequences were aligned using MAFFT. Phylogenetic tree was constructed using the Maximum Likelihood (ML) method with the Whelan and Goldman (WAG) + Freq model with a bootstrap 1,000 replicates in MEGA 6.06 (Tamura et al., 2013).

## Results

### Morphological, cell growth, and cell size features of *A. carterae* and *P. minimum*

**Fig. 1.**
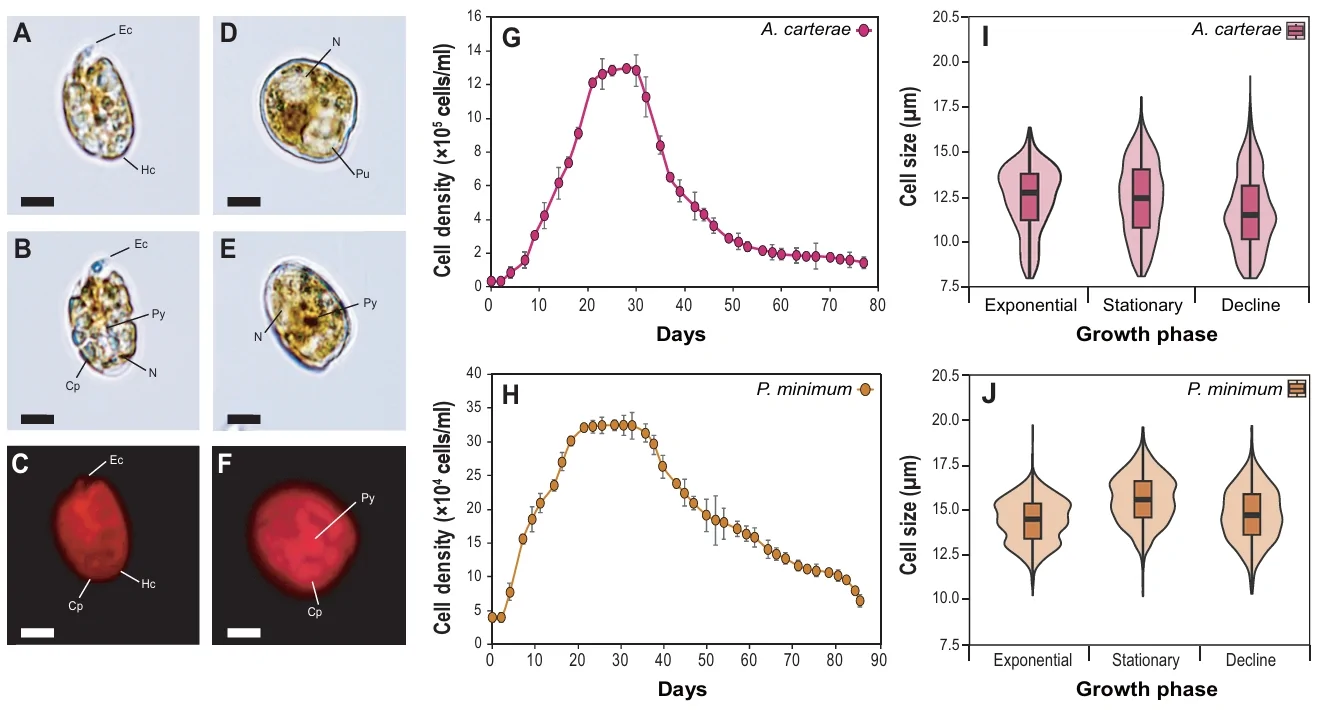
Light and fluorescence microscopy images of the marine dinoflagellates *Amphidinium carterae* (A–C) and *Prorocentrum minimum* (D–F). Ec, epicone; Hc, hypocone; Cp, chloroplast; Py, pyrenoid; Pu, pusule; N, nucleus. Scale bars represent 10 µm. Growth curve and cell size of the marine dinoflagellates *A. carterae* (G–H) and *P. minimum* (I–J).

The marine dinoflagellates *Amphidinium carterae* (D-044) and *Prorocentrum minimum* (D-127) are unarmored and armored species, respectively (Fig. 1A-1F). The morphology of *A. carterae* showed an oval shape and dorsoventrally flattened form. Epicone (Ec) was markedly small and tongue-shaped, and Hypocone (Hc) occupied the majority of the cell volume. The nucleus was spherical and primarily positioned in the posterior region of the HC. Chloroplasts (Cps) were spread across the cells, and a ring-shaped pyrenoid was observed. Also, *P. minimum* (D-127) cells exhibited a typical cordiform shape, enclosed by theca. The nucleus was large and primarily located at the anterior end of the cells. The dark-brown pyrenoid and the plate-shaped Cps were located at the cell center and periphery, respectively.

The strains were cultured under identical conditions at 20°C in f/2 medium to evaluate their growth characteristics, including cell growth patterns (Fig. 1G–1H) and cell size (Fig. 1I–1J). The maximum cell density of *A. carterae* and *P. minimum* peaked at 1.29 × 10^6^ cells/ml (day 28) and 3.13 × 10^5^ cells/ml (day 30), respectively. Commonly, the strains started to proliferate on days 7 to 9 after inoculation, and once they reached the stationary phase, a sharp decrease in cell density was observed during the decline phase. Overall cell size of *A. carterae* was smaller than that of *P. minimum*. In details, *A. carterae* exhibited the largest median cell size during the exponential phase (12.6 μm), which decreased to 12.4 μm during the stationary phase and to 11.7 μm during the decline phase. Also, the median cell size of *P. minimum* was 14.5 μm during the exponential phase and increased to 15.6 μm during the stationary phase. Subsequently, it decreased to 14.7 μm during decline phase.

### Total fatty acid methyl esters (FAME) profile

**Fig. 2.**
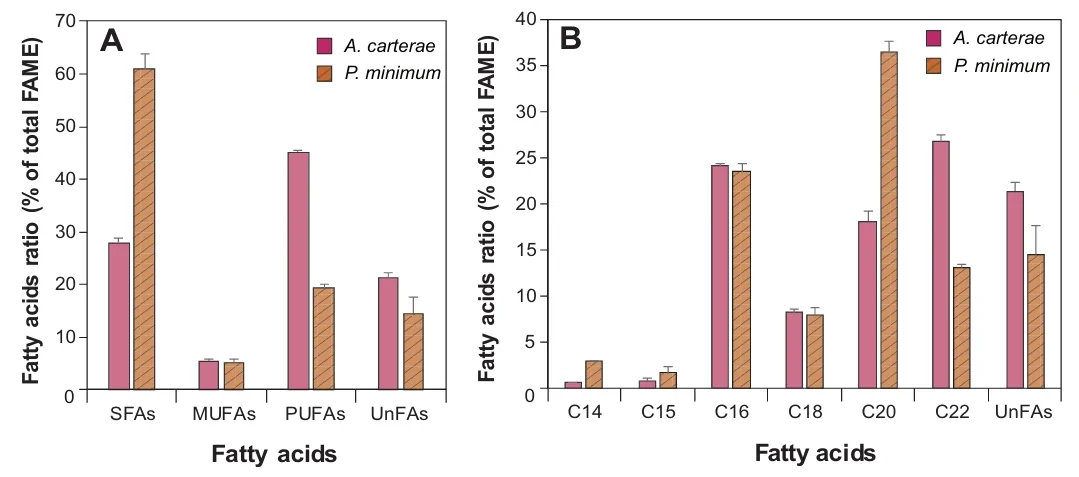
Comparison of fatty acid methyl esters (FAME) compositions (% of total FAME) in the marine dinoflagellates *Amphidinium carterae* and *Prorocentrum minimum*. Percentages of saturated FAs (SFAs), monounsaturated FAs (MUFAs), polyunsaturated FAs (PUFAs), and unidentified FAs (UnFAs) in the total FAs (A). Compositional FMAE profile of total FAs (B).

FAs profile of *A. carterae* and *P. minimum* was analyzed through FAME analysis (Fig. 2, Table 1). The results showed that SFAs and PUFAs accounted for a high proportion in both species, but there were distinct differences in detailed profiles. Specifically, PUFAs were predominant in *A. carterae* (45.0%), while *P. minimum* showed a significantly higher SFA content of 61.0%. In details, palmitic acid (C16:0), which is common to both species, accounted for over 23.5% of total FAs. Among PUFAs, only three omega-3 FAs including alpha-linolenic acid (ALA, C18:3n3), EPA (C20:5n-3), and DHA (C22:6n-3) were detected in both dinoflagellates, and the total concentration in *A. carterae* (67.6 mg/g DW) was much higher than that of *P. minimum* (18.1 mg/g DW). The DHA content of *A. carterae* reached 26.7% of the total FAME profile, approximately double that of *P. minimum* (13.1%). Conversely, *P. minimum* contained the highest percentage of SFAs, mainly arachidic acid (C20:0, 32.6%) and palmitic acid (C16:0, 22.5%). In contrast, omega-6 FAs (C18:2n6, C18:3n6, and C20:4n6) were not detected (N.D.) or detected at very low levels (< 1.9%) in both species.

### Lipid droplets formation depending on the growth stages

**Fig. 3.**
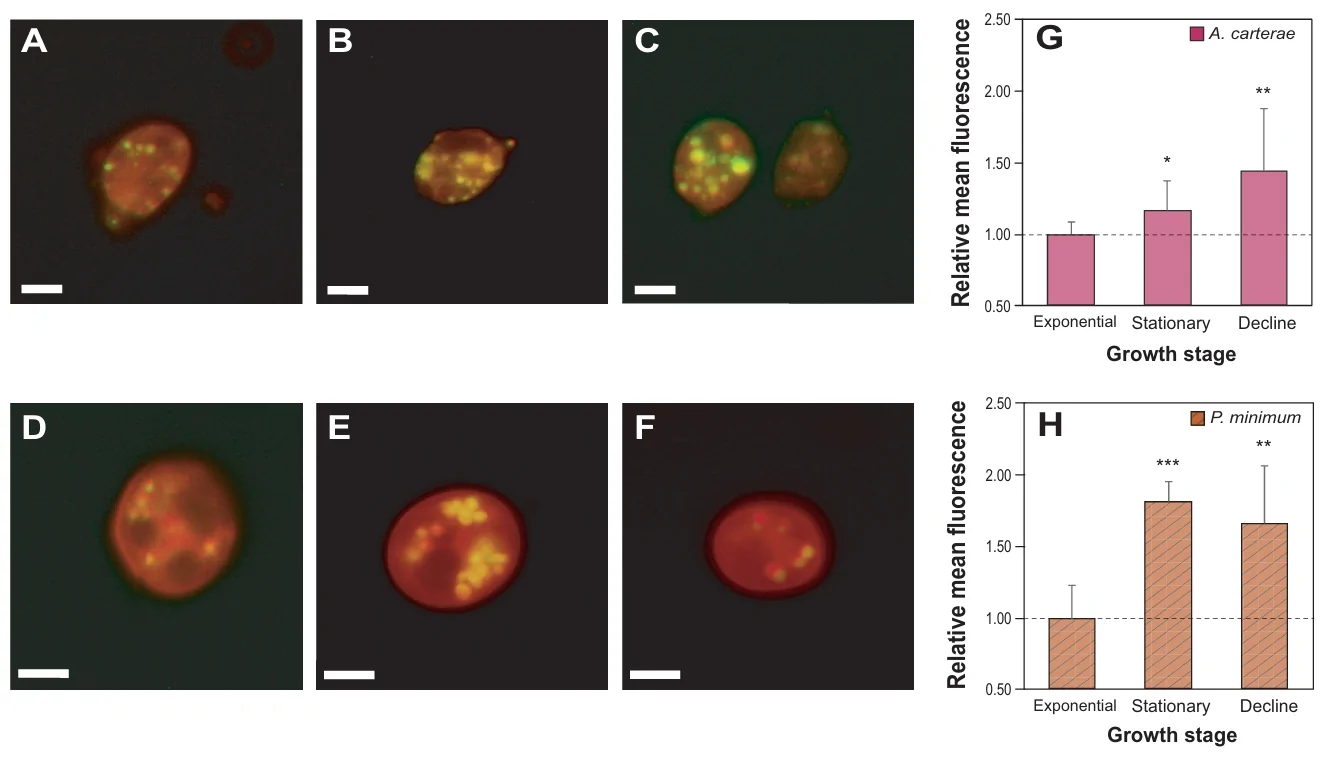
Nile red staining of the marine dinoflagellates *Amphidinium carterae* (A–C) and *Prorocentrum minimum* (D–F). Algal cells with lipid droplets as shown in golden color, and each figure was taken under different growth stages; exponential (A, D), stationary (B, E), and decline phase (C, F). Scale bars represent 10 µm. Relative mean fluorescent levels of lipid droplets in *A. carterae* (G) and *P. minimum* (H) under the different growth stages.

Nile Red staining was performed to observe accumulated neutral lipid in the test species (Fig. 3). The number and size of golden-stained lipid droplets clearly differed depending on the growth phases. In case of *A. carterae*, the relative mean fluorescence intensity increased along with growth stages, reaching up to 1.4-fold higher in the decline phase compared to the exponential phase (*p* < 0.01). In contrast, *P. minimum* peaked a lipid accumulation in the stationary phase (1.8-fold, *p* < 0.001). The fluorescence intensity decreased slightly at the decline phase, while it remained significantly higher than that of the exponential phase (1.6-fold, *p* < 0.01).

### Transcriptome analysis and lipid related GO annotation

Transcriptome analysis yielded 121,969,762 and 126,793,268 raw reads, corresponding to 12.3 and 12.8 Gb nucleotides in *A. carterae* and *P. minimum*, respectively. The total transcripts were clustered into 84,121 and 112,295 transcripts. The transcripts were annotated against the gene ontology (GO) public database (16,066 and 38,233 genes, respectively), and the genes annotated with lipid or FA metabolisms related GO were grouped (Table 2). Overall, *P. minimum* (2,317 genes) contained a larger number of the transcripts and GO-annotated genes than *A. carterae* (1,217 genes). However, the proportion of GO terms related to lipid and FA metabolism was higher in *A. carterae* compared to the total annotated genes. Within the BP category, genes associated with lipid metabolism were 1.8-fold more abundant in *P. minimum* (1,523 genes, 3.98%) than in *A. carterae* (818 genes, 5.09%). Similarly, gene categorized in MF were more numerous in *P. minimum* (779 genes, 1.19%) but showed a higher proportion in *A. carterae* (384 genes, 2.20%). In the cellular component (CC) category, 15 genes annotated with the ‘lipid droplet’ (GO:0005811) were found in both dinoflagellates.

**Fig. 4.**
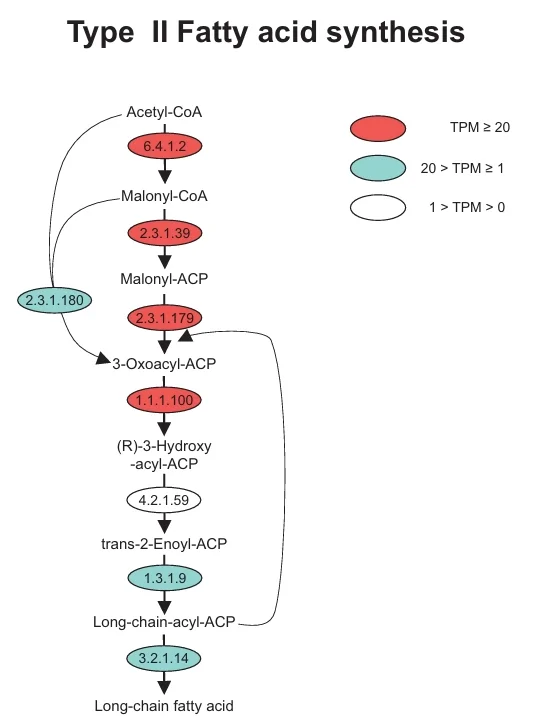
Identification of genes involved in the Type II fatty acid synthesis (FAS II) in marine dinoflagellates *Amphidinium carterae* and *Prorocentrum minimum*. The level of Transcripts Per Million (TPM) is indicated in red, green, and white.

Additionally, we reconstructed the Type II fatty acid synthesis (FAS II) pathway in both dinoflagellates and successfully identified the key enzymes involved in each major stage (Fig. 4). In the initiation stage, acetyl-CoA carboxylase (EC 6.4.1.2) and malonyl-CoA:ACP transacylase (EC 2.3.1.39) exhibited high TPM levels. Within the elongation phase, beta-ketoacyl-ACP synthase (EC 2.3.1.179) and 3-oxoacyl-ACP reductase (EC 1.1.1.100) showed high TPM levels, while beta-ketoacyl-ACP synthase III (EC 2.3.1.180) and enoyl-ACP reductase (EC 1.3.1.9) showed significant TPM levels. For the termination stage, acyl-ACP thioesterase (EC 3.2.1.14), which is responsible for the release of newly synthesized long-chain fatty acids, was also stably expressed.

### Phylogenetic analysis of *LDAH*

**Fig. 5.**
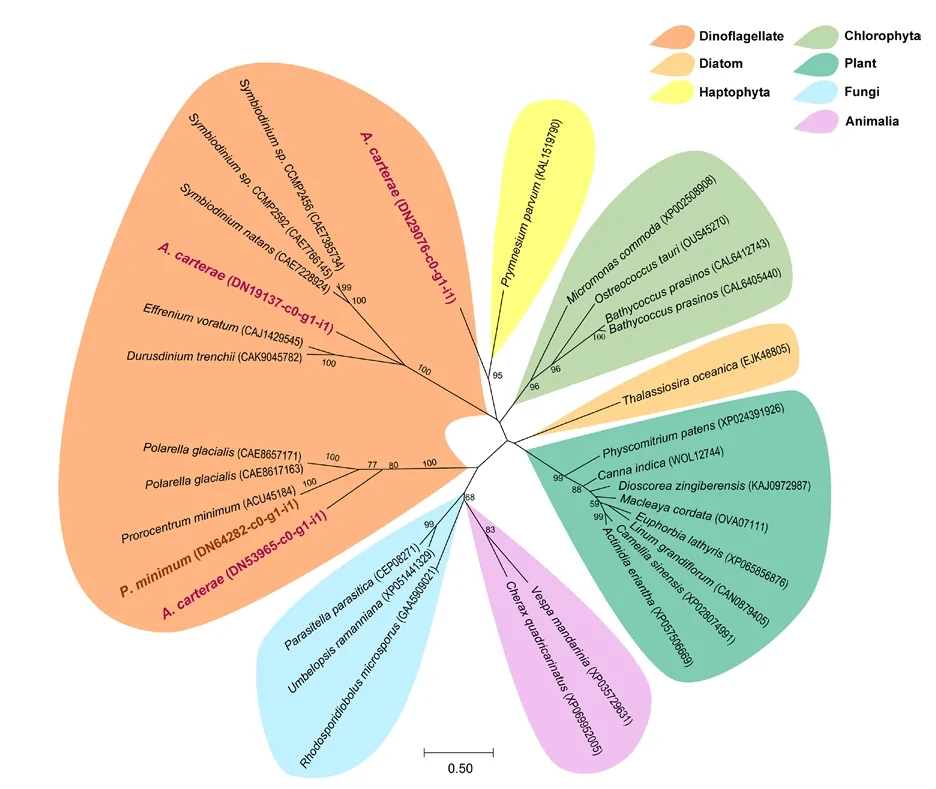
Phylogenetic tree of lipid droplet-associated hydrolase (LDAH) proteins identified in the marine dinoflagellates *Amphidinium carterae* and *Prorocentrum minimum*.

GO analysis revealed a distinct difference in the genes functioning ‘lipid storage’ (GO:0019915) between the species. Four *LDAHs* were identified including one in *P. minimum* (DN64282-c0-g1-i1) and three in *A. carterae* (DN19137-c0-g1-i1, DN29076-c0-g1-i1, and DN53965-c0-g1-i1). Phylogenetic analysis of the selected genes revealed that these belong to the dinoflagellate LDAH lineage or are close to that of haptophyte (Fig. 5). The dinoflagellate LDAHs clade split into two distinct groups: one formed a sister group relationship with the Chlorophyta clade, while the other formed a sister clade with Fungi and Animalia lineages. Meanwhile, the land plant group formed a separate and independent clade, with a high bootstrap support of 99%. Within the dinoflagellate clades, DN19137-c0-g1-i1 from *A. carterae* clustered closely with the sequences from the dinoflagellates *Symbiodinium*. Meanwhile, DN29076-c0-g1-i1 from *A. carterae* was separated from the clade and grouped with the Haptophyta *Prymnesium parvum*, showing 95% of bootstrap support. Another dinoflagellate clade includes DN53965-c0-g1-i1 from *A. carterae* and DN64282-c0-g1-i1 from *P. minimum*, clustering with genes from *P. minimum* and the dinoflagellate *Polarella glacialis*, showing 77% of bootstrap support.

## Discussion

Marine microalgae account for about half of the total oceanic photosynthetic productivity, playing an important role as a foundation of energy production (Beardall and Raven, 2016). Accordingly, microalgal community transfers energy and biomaterials, especially essential FAs, to higher trophic levels such as fish and invertebrates (Falkowski and Raven, 2007). Also, change in FAs composition contribute to regulating cell membrane fluidity and osmotic pressure and maintaining pigments stability, thereby protecting the cells even in extreme environments (Brett and Müller-Navarra, 1997). Several studies have reported that dinoflagellates possess distinct FAs composition, synthesis pathway, and storage form (Remize et al., 2020). Especially, dinoflagellates store higher levels of FAs with enriched PUFAs compared to other microalgae. Of them, the genera *Amphidinium* and *Prorocentrum* are known to accumulate high concentrations of omega-3 (Jaeckisch et al., 2011; Řezanka et al., 2017).

In the present study, the total FAs yield of *A. carterae* was 67.6 mg/g DW, more than 3.7 times higher than that of *P. minimum* (18.1 mg/g DW), showing a clear advantage in the productivity. FAs yield of *A. carterae* was similar to that of industrial strains *Nannochloropsis oculata* (50–60 mg/g DW) (Ma et al., 2016) and *Isochrysis galbana* (40–45 mg/g DW) (Ryckebosch et al., 2012). Regarding PUFAs, consistent with our results, the PUFA content of *A. carterae* was reported to be as high as 53.2%, with DHA and EPA accounting for 30.6% and 19.7%, respectively (Mendoza-Flores and Sánchez-Saavedra, 2023). These values are significantly higher than those reported for other dinoflagellates. For example, DHA levels of only 8–23% have been observed in marine gymnodinioid dinoflagellates (*Karenia*, *Karlodinium*, and *Takayama*) (Mooney et al., 2007). Also, *Prorocentrum* micans was reported to contain 22.8% of DHA and 13.1% of EPA, both of which were higher than those of *P. minimum* in the present study (Suh et al., 2015). Furthermore, while PUFAs accounted for up to 60% of total FA in marine green algae *Tetraselmis suecica* and diatom *Phaeodactylum tricornutum*, but their DHA remained minimal ranging from 0.2 to 1.7% (Qiao et al., 2016; Volkman et al., 1989). These findings demonstrate that although total FA yield and PUFA profiles vary by species and strains (Vazhappilly and Chen, 1998), dinoflagellates nevertheless showed a remarkable capacity for PUFA production.

Recent research on dinoflagellates has focused on transcriptome analysis due to their enormous and condensed genomic structure (Bui et al., 2025; Muhammad et al., 2025; Wang et al., 2021b). Number of functional genes were identified and their molecular responses were elucidated under various environmental stressors (Abassi and Ki, 2022; Abassi et al., 2023), while only a few studies have analyzed FAs related genes. In this regard, we identified genes participating in the lipid and FAs metabolisms through transcriptome analysis in the two marine dinoflagellates. Through GO analysis, the number of transcripts, 1,217 in *A. carterae* and 2,317 in *P. minimum*, were identified. In other cases, a study on *Heterocapsa bohaiensis* revealed significant changes in transcripts after treatment with allelochemicals, and the key gene groups involved in PUFA production (Acetyl-CoA carboxylase, FA desaturase, and elongase) were significantly up-regulated (Bachvaroff et al., 2014). Also, Kalinina et al. (2025) reported that expression of FA desaturase was suppressed under phosphorus-limited conditions, accompanied by a decrease in UFAs production in *Prorocentrum cordatum*. These results suggest that the FA metabolism responds sensitively to environmental changes (Chang et al., 2026; Thiyagarajan et al., 2020). In this regard, comparative transcriptomic analysis among dinoflagellates revealed species-specific differences in the number of FAs-associated genes and their gain/loss patterns (Stephens et al., 2021). Consistently, Rein et al. (2025) reported that while *K. brevis* possesses a high number of isoforms, proportion of core FA gene families was relatively low compared to others. This aligns with our findings, where the proportion of FAs related genes was markedly lower in *P. minimum*. These suggest that *P. minimum* contains a larger transcript due to various isoforms and gene replication, whereas *A. carterae* exhibits a more enriched genetic profile in terms of FA metabolisms.

When analyzing GO terms related to FAs metabolism, we focused on lipid storage (GO:0019915) category. Since PUFAs are stored in LD in the form of triglycerides (TAG) as well as cell membranes, genes participating in LD formation and maintenance also play an important role as lipid regulators (Danielli et al., 2023). In this regard, LDAH plays a crucial role in maintaining lipid homeostasis by regulating the balance between FA degradation and storage within LD (Wilfling et al., 2014). Although LDAH possesses a hydrolase domain, current research has suggested that it promotes LD fusion and induces intracellular TAG accumulation rather than hydrolysis (Goo et al., 2014, 2017; Kong et al., 2018; Kory et al., 2017). In details, biochemical analyses showed that LDAH displayed weak cholesterol ester hydrolase activity but little TAG hydrolase activity (Goo et al., 2014, 2017). The enzyme and coding genes were primarily identified in model land plants (Zhao et al., 2025), where the presence of LDAH-like proteins was first identified in the LD of the marine protist *Aurantiochytrium limacinum* (Yoneda et al., 2022). Interestingly, we identified three distinct *LDAHs* in *A. carterae*, whereas only a single gene was identified in *P. minimum*. Notably, phylogenetic analysis revealed that the *LDAHs* had a distinct evolutionary origin, implying an evolutionary strategy that enables them to gain functional flexibility for environmental adaptation. Indeed, Goold et al. (2015) also reported that various microalgal species possess evolutionarily unique LD-associated protein families, which perform complementary roles in lipid redistribution, storage, and release. The present study analyzed FA profiles and associated transcripts to identify *LDAHs* in dinoflagellates for the first time. Furthermore, the multigenic LDAH system of *A. carterae* can be utilized in metabolic engineering research to improve lipid productivity and/or storage in microalgae with industrial potential. In this regard, functional studies are needed to experimentally verify the enzymatic role and specific regulatory mechanisms of LDAH system in marine dinoflagellates.

## Conclusion

The present study provides a molecular and biochemical characterization of FA metabolism in two marine dinoflagellates *A. carterae* and *P. minimum*. Through Nile red-based LD observation and GC-FAME analyses, we demonstrated that *A. carterae* possesses a superior capacity for omega-3 (DHA and EPA) biosynthesis and lipid storage. Also, transcriptomic analysis revealed an enriched genes associated with lipid and FAs metabolism in *A. carterae*, providing a molecular basis for its robust biosynthetic potential. The three *LDAHs* identified in *A. carterae* suggest significant gene diversification that is likely to contribute to lipid storage and related metabolic flexibility. Collectively, these findings imply that *A. carterae* harbors an enhanced and flexible genetic system for lipid production and highlight that *A. carterae* and *LDHA* are noteworthy candidates for blue biotechnology applications and genetic engineering research.

## Figures and Tables

**Table 1.** Comparison of fatty acid yields (mg/g DW and % DW) and FAME profiles (%) in marine dinoflagellates *Amphidinium carterae* and *Prorocentrum minimum*. DW, dry weight; FAME, fatty acid methyl esters; N.D., not detected. Values are expressed as Mean ± SD (n = 3).

Contents	FAs yield (mg/g DW)		FAs yield (DW %)		FAME profile (%)	
	*A. carterae*	*P. minimum*	*A. carterae*	*P. minimum*	*A. carterae*	*P. minimum*
C14:0	1.0 ± 0.1	3.0 ± 0.5	0.1 ± 0.0	0.3 ± 0.1	0.6 ± 0.0	2.9 ± 0.1
C14:1	N.D.	N.D.	N.D.	N.D.	N.D.	N.D.
C15:1	1.1 ± 0.5	1.6 ± 0.7	0.1 ± 0.0	0.2 ± 0.1	0.8 ± 0.3	1.7 ± 0.8
C16:0	35.8 ± 3.6	23.4 ± 3.6	3.6 ± 0.4	2.3 ± 0.4	23.3 ± 0.4	22.5 ± 0.8
C16:1	1.3 ± 0.2	1.1 ± 0.2	0.1 ± 0.0	0.1 ± 0.0	0.8 ± 0.1	1 ± 0.0
C18:0	4.1 ± 0.3	3.1 ± 0.5	0.4 ± 0.0	0.3 ± 0.1	2.7 ± 0.2	3.1 ± 0.8
C18:1 cis n9	6.1 ± 1.6	2.1 ± 0.5	0.6 ± 0.2	0.2 ± 0.0	3.9 ± 0.5	2 ± 0.1
C18:1 trans n9	N.D.	N.D.	N.D.	N.D.	N.D.	N.D.
C18:2 cis n6	N.D.	0.1 ± 0	N.D.	N.D.	N.D.	0.1 ± 0.0
C18:2 trans n6	2.0 ± 0.3	1.8 ± 0.4	0.2 ± 0.0	0.2 ± 0.0	1.3 ± 0.1	1.7 ± 0.1
C18:3n6	N.D.	0.1 ± 0.0	N.D.	N.D.	N.D.	0.1 ± 0.0
C18:3n3	0.6 ± 0.1	0.9 ± 0.1	0.1 ± 0.0	0.1 ± 0.0	0.4 ± 0.1	0.9 ± 0.1
C20:0	2.0 ± 0.2	33.9 ± 5.1	0.2 ± 0.0	3.4 ± 0.5	1.3 ± 0.2	32.6 ± 1.3
C20:1n9	N.D.	0.5 ± 0.1	N.D.	N.D.	N.D.	0.5 ± 0.0
C20:2	N.D.	0.1 ± 0.1	N.D.	N.D.	N.D.	0.1 ± 0.0
C21:0	N.D.	N.D.	N.D.	N.D.	N.D.	N.D.
C20:3n6	N.D.	N.D.	N.D.	N.D.	N.D.	N.D.
C20:4n6	N.D.	N.D.	N.D.	N.D.	N.D.	N.D.
C20:3n3	N.D.	N.D.	N.D.	N.D.	N.D.	N.D.
C20:5n3	25.7 ± 1.2	3.5 ± 1.0	2.6 ± 0.1	0.3 ± 0.1	16.8 ± 1.0	3.3 ± 0.4
C22:0	0.2 ± 0.2	N.D.	N.D.	N.D.	0.1 ± 0.2	N.D.
C22:1n9	N.D.	N.D.	N.D.	N.D.	N.D.	N.D.
C23:0	N.D.	N.D.	N.D.	N.D.	N.D.	N.D.
C22:6n3	41.3 ± 6.1	13.7 ± 2.9	4.1 ± 0.6	1.4 ± 0.3	26.7 ± 0.8	13.1 ± 0.4
C24:1	N.D.	N.D.	N.D.	N.D.	N.D.	N.D.
Unidentified FAs	33.1 ± 5.1	15.7 ± 6.3	3.3 ± 0.5	1.6 ± 0.6	21.4 ± 1.0	14.5 ± 3.2

**Table 2.** Gene Ontology (GO) classification of lipid and fatty acid metabolism-related genes identified from marine dinoflagellates *Amphidinium carterae* and *Prorocentrum minimum*. The percentages of each GO category represent the ratio of the number of genes selected within the category. N.D., not detected.

GO category	Top GO	GO term	No. genes in *A. carterae*	No. genes in *P. minimum*
Biological process	GO:0016042	lipid catabolic process	170	220
	GO:0006631	fatty acid metabolic process	191	240
	GO:0046486	glycerolipid metabolic process	155	285
	GO:1901568	fatty acid derivative metabolic process	10	36
	GO:0008610	lipid biosynthetic process	48	139
	GO:0006644	phospholipid metabolic process	17	62
	GO:0006643	membrane lipid metabolic process	104	214
	GO:0006869	lipid transport	43	168
	GO:1903509	liposaccharide metabolic process	40	34
	GO:0008202	steroid metabolic process	18	91
	GO:0030258	lipid modification	3	19
	GO:0055091	phospholipid homeostasis	N.D.	3
	GO:0006720	isoprenoid metabolic process	11	10
	GO:0019216	regulation of lipid metabolic process	2	1
	GO:0019915	lipid storage	6	1
			5.09%	3.98%
Cellular component	GO:0005811	lipid droplet	15	15
			0.10 %	0.05 %
Molecular function	GO:0016407	acetyltransferase activity	67	85
	GO:0016705	oxidoreductase activity, acting on paired donors, with incorporation or reduction of molecular oxygen	205	587
	GO:0120515	fatty acid-CoA ligase activity	33	39
	GO:0004312	fatty acid synthase activity	71	59
	GO:0009922	fatty acid elongase activity	8	9
			2.20%	1.19%

## Data Availability

The datasets presented in this study can be found in online NCBI Sequence Read Archive (SRA) database (PRJNA14639770).
